# Demographic profile, treatment patterns, and healthcare resource use of Mild Cognitive Impairment and Alzheimer's Disease in Taiwan

**DOI:** 10.1002/alz70860_099583

**Published:** 2025-12-23

**Authors:** Chaur‐Jong Hu, Yao Hsien Huang, Bruce CM Wang, Sarah Cho, Shih‐Pei Shen, Rachel Newson, Chao‐Hsiun Tang

**Affiliations:** ^1^ Taipei Medical University, Taipei, Taiwan; ^2^ Eli Lilly and Company, Taipei, Taiwan; ^3^ Elysia Group Ltd, Taipei, Taiwan; ^4^ Eli Lilly and Company, Sydney, Australia

## Abstract

**Background:**

There is limited information on the epidemiology of MCI and Alzheimer's Disease in Taiwan. As such the current study sought to describe the demographic profiles, healthcare resource use, and trends in treatment patterns for mild cognitive impairment (MCI) and Alzheimer's disease (AD) in Taiwan.

**Method:**

Data was analysed using the National Health Insurance Research Database (NHIRD), a database covering 99.6% of the population. An annual analysis was conducted from 2018 to 2022 and included people aged ≥50 years diagnosed with MCI (ICD‐9: 331.83; ICD‐10: F067) or AD (ICD‐9: 331.0; ICD‐10: G30.x). As there is no standard coding for AD severity, a diagnostic algorithm was developed based on literature, NHIRD feasibility and neurologist expert consultation. On this basis patients were stratified by disease severity (MCI, mild AD, moderate AD, severe AD). Descriptive statistics summarized demographic characteristics, annual treatment trends, and healthcare resource use.

**Result:**

From 2018 to 2022, the prevalence of people with AD increased by 19.6%, reaching 131,053 in 2022. Females represented approximately two‐thirds of the AD population. In terms of treatment, Donepezil use rose from 20.3% in 2018 to 28.2% in 2022, with the highest usage among patients with mild AD (74.6% in 2022). Memantine use increased notably among moderate (64.7%) and severe AD patients (54.3%), reflecting its role in later‐stage management. Traditional Chinese Medicine (TCM) utilization remained consistent, peaking at 22.2% in 2022. Severe AD patients exhibited high rates of non‐pharmacological therapies such as physical therapy (5.3%), which were rarely used in mild AD cases. Patients with MCI demonstrated limited pharmacological intervention, with stable prevalence at 24,440 in 2022 and minimal treatment variation. Differences in age distribution showed a high median age in severe AD patients (84 years) relative to mild (81 years) and moderate (81 years). Moderate and severe AD patients had a high reliance on combination therapies involving cholinesterase inhibitors and memantine. Healthcare resource use was noteworthy.

**Conclusion:**

The results highlight increasing AD prevalence and evolving treatment patterns, with rising use of donepezil in MCI and memantine in advanced stages. These trends can inform healthcare strategies for managing MCI and AD in Taiwan.

